# EXPLORING PERCEPTIONS OF A SOCIALLY ASSISTIVE ROBOT IN ASSISTED LIVING: RECOMMENDATIONS FROM FAMILY CARE PARTNERS

**DOI:** 10.1093/geroni/igad104.3736

**Published:** 2023-12-21

**Authors:** Shannon Power, Kasey Smith, Lydia Burton, Reuben Abedine, Jenay Beer, Anne Adams

**Affiliations:** University of Georgia, Athens, Georgia, United States; SimpleC, Atlanta, Georgia, United States; Institute of Gerontology, UGA, Athens, Georgia, United States; The University of Georgia, Athens, Georgia, United States; University of Georgia, Athens, Georgia, United States; SimpleC, LLC, Atlanta, Georgia, United States

## Abstract

Research has shown that socially assistive robots are effective in delivering health and behavioral interventions to persons with and at-risk for dementia living in assisted living facilities. However, to gain a more comprehensive understanding on the potential benefits of socially assistive robots, family care partners should also be consulted for design recommendations. Thus, we conducted semi-structured virtual focus groups to investigate family care partners’ (N=10) perceived benefits, concerns, and design recommendations of a socially assistive robot for their loved ones with or at-risk for dementia. Robot interactions (e.g., activity reminders, trivia entertainment, health prompts) were presented to participants via video, followed by in-depth questions and discussion. Thematic qualitative analysis (intercoder agreement = 87.5%) resulted in most recommendations on usefulness and function (30%), followed by engagement and entertainment (18%), social attributes (15%), customization (14%), health promotion, education, and behavior (9%), appearance (7%), and other (7%). Family care partners recommended improvements to build interactivity, social connection, and usefulness, such as improvements to robot appearance, customization and adaptability in robot capability that matches disease progression, and considerations for ethical implementation (i.e., the robot as supplement, not replacement, of human caregiving). Our findings represent the personal perspective of care partners intimately acquainted with dementia and caregiving, and from this perspective, yield specific recommendations for useful design of socially assistive robots.

